# CLCf is an endosomal resident proton/chloride antiporter during salt stress

**DOI:** 10.1093/plphys/kiaf145

**Published:** 2025-04-24

**Authors:** Daniel W McKay, Melanie Krebs, Stefanie Wege, Michelle Uebele-Pérez, Upendo Lupanga, Karin Schumacher

**Affiliations:** Cell Biology, Centre for Organismal Studies (COS), Heidelberg University, Im Neuenheimer Feld 230, 69120 Heidelberg, Germany; Cell Biology, Centre for Organismal Studies (COS), Heidelberg University, Im Neuenheimer Feld 230, 69120 Heidelberg, Germany; Institute of Crop Science and Resource Conservation (INRES), Rheinische Friedrich-Wilhelms Universität Bonn, D-53113 Bonn, Germany; Cell Biology, Centre for Organismal Studies (COS), Heidelberg University, Im Neuenheimer Feld 230, 69120 Heidelberg, Germany; Cell Biology, Centre for Organismal Studies (COS), Heidelberg University, Im Neuenheimer Feld 230, 69120 Heidelberg, Germany; Cell Biology, Centre for Organismal Studies (COS), Heidelberg University, Im Neuenheimer Feld 230, 69120 Heidelberg, Germany

## Abstract

CHLORIDE CHANNEL f (CLCf) has a role in maintaining *trans*-Golgi Network/Early Endosome function under all conditions and is unlikely to mediate plasma membrane chloride transport during salt stress.

Dear Editor,

Chloride is an essential micronutrient for plants, which can have beneficial effects when present at macronutrient levels while excess chloride, as found in saline soils, often has negative impact on plant biomass and especially crop yield (Wege et al. 2017). A reduction of shoot chloride content often positively associates with increased salt stress tolerance and biomass production, particularly a reduction in foliar chloride content (Wu and Li 2019). Shoot chloride content can be regulated by restricting chloride translocation through the root vasculature, for example, by excluding chloride from root epidermal and cortex cells. Chloride that has entered root cells might therefore be exported out of the cell through a plasma membrane (PM) localized chloride selective export protein to regulate chloride content in plants. Different protein families in plants can mediate chloride transport, among them are members of the Chloride Channel (CLC) family. CLC proteins, however, typically localize in endomembrane compartments and not the PM where they could directly mediate chloride export. Chloride transport proteins are crucial in endomembrane compartments as, in addition to the role of chloride in the oxygen evolving complex, stomatal aperture regulation, and turgor-driven cell expansion, chloride is utilized as a countercharge in cellular compartments (Wege et al. 2017). As such, chloride plays a major role in compartments such as the *trans*-Golgi Network/Early Endosome (TGN/EE), an important trafficking hub in plant cells. The transport circuits and regulation that enable chloride to play this role, however, are not yet fully elucidated. In total, Arabidopsis (*Arabidopsis thaliana*) has 7 CLCs. CLCa, CLCb, CLCc, and CLCg are found at the tonoplast, CLCd and CLCf in the TGN/EE and CLCe at the thylakoid membrane (De Angeli et al. 2006; Marmagne et al. 2007; von der Fecht-Bartenbach et al. 2007; Jossier et al. 2010; von der Fecht-Bartenbach et al. 2010; Nguyen et al. 2016). CLCd and CLCf are both ubiquitously expressed and appear to compensate the others function, yet, CLCd and CLCf belong to different subgroups of the CLC family and did not arise from a duplication event (Scholl et al. 2021). Therefore, these proteins either evolved to perform redundant roles in plants or these proteins have nonredundant roles that are yet to be identified. Double knockouts are not viable even under standard, nonstressed conditions (von der Fecht-Bartenbach et al. 2007; Scholl et al. 2021).

Recently, Rajappa et al. (2024) investigated the role of CLCf under salt stress and investigated a potential role for the protein in reducing accumulation of chloride in plants under salt stress. The authors propose that CLCf translocates to the PM of root epidermal cells under salt stress to efflux chloride from roots. Rajappa et al. (2024) found that *CLCf* gene expression was upregulated after treatment of plants with 100 mm NaCl and report that the primary root growth of *clcf* knockout plants is hypersensitive to salt when grown on media containing 50 mm NaCl. A potential role of CLCf as a PM chloride antiporter is suggested, with a potential translocation of green fluorescent protein (GFP) tagged CLCf from the TGN/EE to the PM observed after 4 and 6 h treatments with 100 mm NaCl using heterologous expression in *Nicotiana benthamiana* and Arabidopsis leaf protoplasts. Localization analysis of stably transformed Arabidopsis plants expressing *35S:GFP-CLCf* was also performed. The images presented for the unstressed and NaCl stressed conditions were taken from different optical sections of root epidermal cells, which can lead to ambiguous results when inferring subcellular localization, making interpretation challenging (Guo et al. 2023). In cortical optical sections, TGN/EE puncta can be seen across the surface of the cell, whereas in optical sections from the center of the cell, the large vacuole displaces the TGN/EE toward the cell periphery (Guo et al. 2023). In addition, as is often the case with N-terminal GFP-tagging, faint cytosolic background fluorescence can be observed, which can give the impression of a PM signal in single optical sections. The images presented by Rajappa et al. (2024) therefore do not fully allow conclusion regarding plasma membrane localization of GFP-CLCf under both control and NaCl-treated conditions.

The identification of a PM localized proton/chloride antiporter would substantially alter our current understanding of cellular chloride homeostasis and would provide an outstanding candidate for salinity tolerance research. Therefore, we aimed to clarify the localization of CLCf under control and salt stress conditions by performing a careful re-evaluation of the subcellular localization of fluorescently labeled CLCf in Arabidopsis root cells.

CLCf tagged with monomeric red fluorescent protein (mRFP), was previously shown to colocalize with the V-ATPase subunit, VHA-a1, and the anion/proton antiporter CLCd at the TGN/EE (Scholl et al. 2021). This was corroborated by Rajappa et al. (2024) using GFP-CLCf under control conditions but PM localization was observed after a NaCl shock. To assess the suggested salt-induced TGN/EE to PM translocation of CLCf, localization analysis of CLCf-GFP was performed in root epidermal cells that were either grown constitutively on 75 mm NaCl (salt stress) or transferred to medium containing 75 mm NaCl, 6 h before imaging (salt shock). Under both treatments, CLCf-GFP remained exclusively localized in TGN/EE-like puncta in the elongation zone (Fig. A, Supplementary Fig. S1) and in mature epidermal cells (Fig. B, Supplementary Fig. S1). Furthermore, overlay of CLCf-GFP signal and the membrane tracing dye, FM4-64, revealed a clear separation of the 2 signals, arguing against a TGN/EE to PM translocation of CLCf during exposure to NaCl. Thus, our results provide no evidence that CLCf translocates from the TGN/EE to the PM under either sudden salt exposure nor under prolonged salt stress. Therefore, we propose a cellular role for CLCf exclusively within endomembrane compartments.

**Figure 1.**
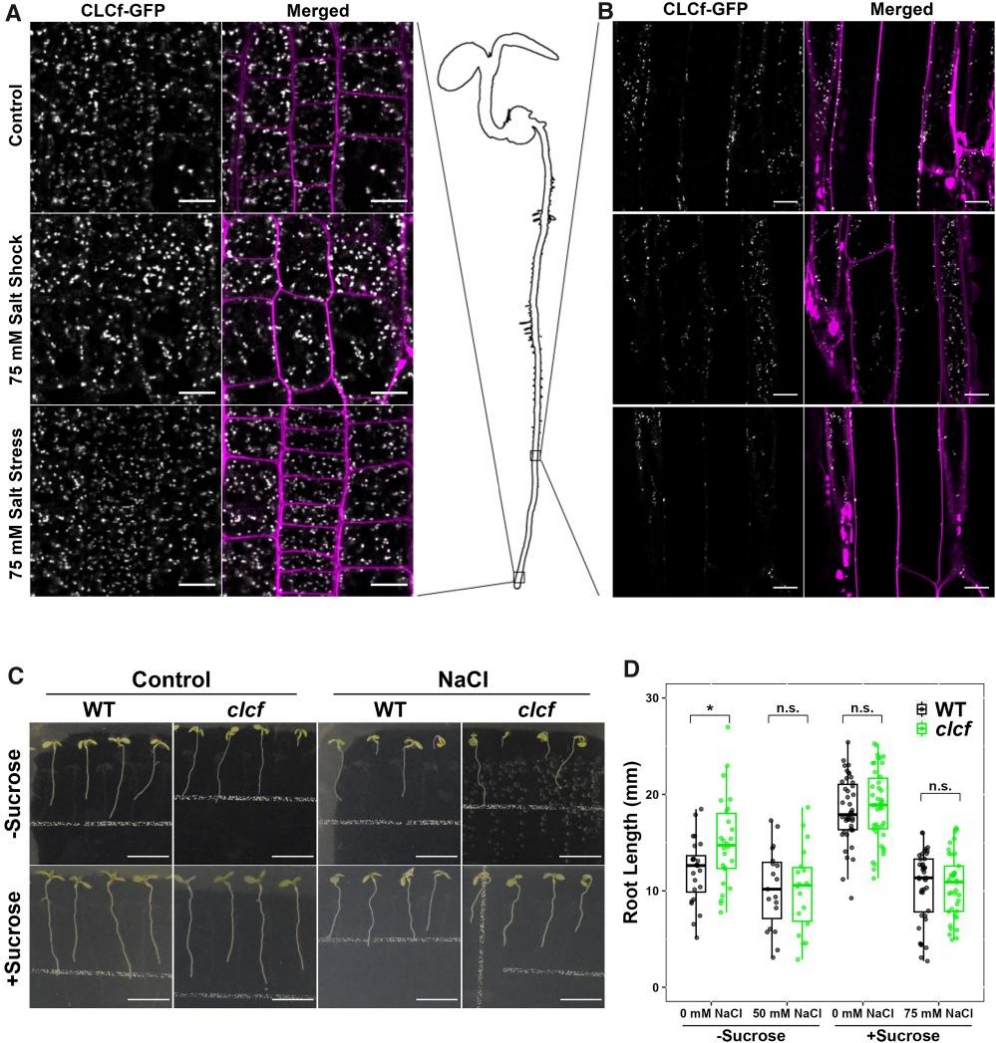
CLCf is a TGN/EE resident protein that is not critical to maintaining root growth under moderate salt stress. **A** and **B)** CLSM analysis shows that CLCf-GFP (white) localizes to TGN/EE-like puncta in root epidermal cells of both the elongation zone **(A)** and in mature root cells **(B)** in 9-day old seedlings after treatment with 75 mm NaCl for 6 h (Salt Shock) or when constitutively grown on media containing 75 mm NaCl (Salt Stress). CLCf-GFP puncta do not colocalize with FM4-64 labelled PM (merged). All images are a single layer of image stacks (Supplementary Fig. S1). Scale bars = 10 *µ*m. **C** and **D)** Primary root length of *clcf* mutants is not affected when grown for 1 week on media containing salt. Plants were either grown on full strength MS, phytagel media without sucrose, with or without 50 mm NaCl (−Sucrose) or on 1/2 strength MS, phyto agar media, with sucrose with or without 75 mm NaCl (+Sucrose). The upper white line is part of the background equipment. Scale bars = 10 mm. Statistical significance was determined via *t*-test. *<0.05 <n.s. For the boxplots, center line = median, box limits = upper and lower quartiles, whiskers = range to a maximum of 1.5× the interquartile range, points = individual data points.

To further address the putative enrolment of CLCf in salt tolerance, we aimed to confirm the salt hypersensitivity of *clcf* mutants using the first published mutant allele for *clcf* (SALK_112962; Scholl et al. 2021). To rule out conditional effects resulting from different medium compositions, we measured primary root length of 1-week-old seedlings grown on our standard medium consisting of 1/2 strength Murashige and Skoog (MS), 0.5% sucrose, and 0.8% phyto agar (Duchefa) with a salt treatment of 75 mm NaCl in addition to measurements from plates containing the medium used by Rajappa et al. (2024), which consisted of full strength MS, no added sucrose, and 0.5% Gelrite with a salt treatment of 50 mm NaCl. When grown on the full-strength MS media without sucrose, germination of seeds was reduced and seedlings which failed to develop past germination were frequently encountered for both genotypes. Seedlings which failed to develop were excluded from analysis, however, more consistent results were still obtained with media using 1/2 strength MS with the addition of sucrose. For both media compositions, however, *clcf* root length did not significantly differ from wildtype (WT) when grown on 50 mm or 75 mm NaCl (Fig. C and D). We also do not observe a salt hypersensitive root growth defect in older *clcf* seedlings, grown to 2 weeks, when compared with WT plants, whereas we were able to confirm the previously characterized salt hypersensitivity of the TGN/EE potassium/proton double knockout mutant, *nhx5/nhx6* (Supplementary Fig. S2; Bassil et al. 2011). In summary, we did not observe hypersensitivity to NaCl at the level of primary root growth of this previously characterized *clcf* mutant allele.

Here, we corroborate the previous finding that CLCf is a TGN/EE resident chloride/proton antiporter, where it is suggested to assist in the regulation of chloride concentrations in the TGN/EE, which are critical for pH control (Sze and Chanroj 2018; Scholl et al. 2021; Rajappa et al. 2024). Furthermore, we show that CLCf remains in the TGN/EE in root epidermal cells during short- and long-term exposure to salt stress, which is in agreement with its proposed role in regulating TGN/EE chloride content, a role that is still required during salt stress. The fact that we were not able to observe hypersensitivity of *clcf* to NaCl under the selected conditions does not necessarily contradict a putative role for CLCf in salt tolerance. Therefore, it will be of interest to further investigate an involvement of CLCf during salt stress by specifically focusing on its role for TGN/EE ion regulation. While chloride concentrations in the TGN/EE cannot yet be directly measured, genetically encoded pH indicators enable noninvasive pH measurements with high spatial and temporal resolution. Dissecting anion and pH dynamics in the TGN/EE and other endomembrane compartments in *clcf* or higher order transport mutants when exposed to salt stress will give valuable insight in the mechanisms that govern salt tolerance on a cellular level.

CLCf was observed to translocate under salt stress conditions when transiently expressed in *N. benthamiana* and Arabidopsis protoplasts but translocation was not observed when stably expressed in Arabidopsis. These contrasting results highlight the importance of caution when interpreting localization results in heterologous systems. Previous reviews highlight the risks of transient expression where selection of highly expressing cells can result in an overabundance of a protein, saturating receptors or other binding proteins that may be required for trafficking or the formation of localization determining heterodimers (North 2006; Guo et al. 2023). Furthermore, the size of vacuoles in plant cells can result in displacement of the cytoplasm to the cell peripheries where it can be as thin as 1 *µ*m, creating difficulties in determining what is cytoplasmic and what is PM (Guo et al. 2023). Examples of this can be seen in the images of mature root epidermal cells (Fig. B), where images contain adjacent cells captured across the cortical or middle slice and therefore have TGN/EE puncta across the surface of the cell or around the cell border, respectively. Due to these potential issues, it is important to confirm localization in native tissue where protein abundance is better regulated and to ensure that images are taken from the same optical layer of the cell so that correct comparisons can be made and localization is not misrepresented.

Chloride homeostasis is crucial for various cellular processes and imbalances, especially excess chloride, potentially compromising plant biomass production. Therefore, the identification and characterization of mechanisms that control cellular chloride homeostasis could be essential for developing new crop traits that are more resilient toward chloride stress. While understanding the regulation of chloride transport across the PM is important due to the function of the PM as a cellular barrier, a complete understanding of cellular chloride homeostasis requires consideration of all transport mechanisms that contribute to the flux and compartmentalization of the nutrient from the subcellular to the tissue level. This work, alongside with other recent studies, advances our understanding of chloride transport at the TGN/EE, where chloride transporters such as CLCd, CLCf, and CATION CHLORIDE COTRANSPORTER1 (CCC1) have been identified and partially characterized (Colmenero-Flores et al. 2007; Marmagne et al. 2007; von der Fecht-Bartenbach et al. 2007; Henderson et al. 2015; Sze and Chanroj 2018; Scholl et al. 2021; McKay et al. 2022; Rajappa et al. 2024). However, future research on cellular chloride homeostasis must not only identify the components responsible for chloride transport across individual cellular membranes, but also elucidate how distinct transport systems across these different membranes collectively contribute to regulate chloride concentrations at the whole-plant level.

## Supplementary Material

kiaf145_Supplementary_Data

## Data Availability

The data underlying this article will be shared on reasonable request to the corresponding author.

## Supplementary data

The following materials are available in the online version of this article.

**Supplementary Figure S1.** CLCf-GFP localizes to puncta in root cells under control and salt stress conditions.

**Supplementary Figure S2.** CLCf is not required for maintaining root growth of seedlings during moderate salt stress.

## Dive Curated Terms

The following phenotypic, genotypic, and functional terms are of significance to the work described in this paper:

chloride CHEBI: CHEBI:17996

CLSM Gramene: confocal laser scanning microscopy
CLSM Araport: confocal laser scanning microscopy
