# Supplementary material for: CLCf is an endosomal resident proton/chloride antiporter during salt stress
Source: Plant Physiol. 2025 Apr 24;197(4):kiaf145. doi: 10.1093/plphys/kiaf145 (PMC12018876; doi:10.1093/plphys/kiaf145)
Supplement: kiaf145_Supplementary_Data [file kiaf145_supplementary_data.pdf]

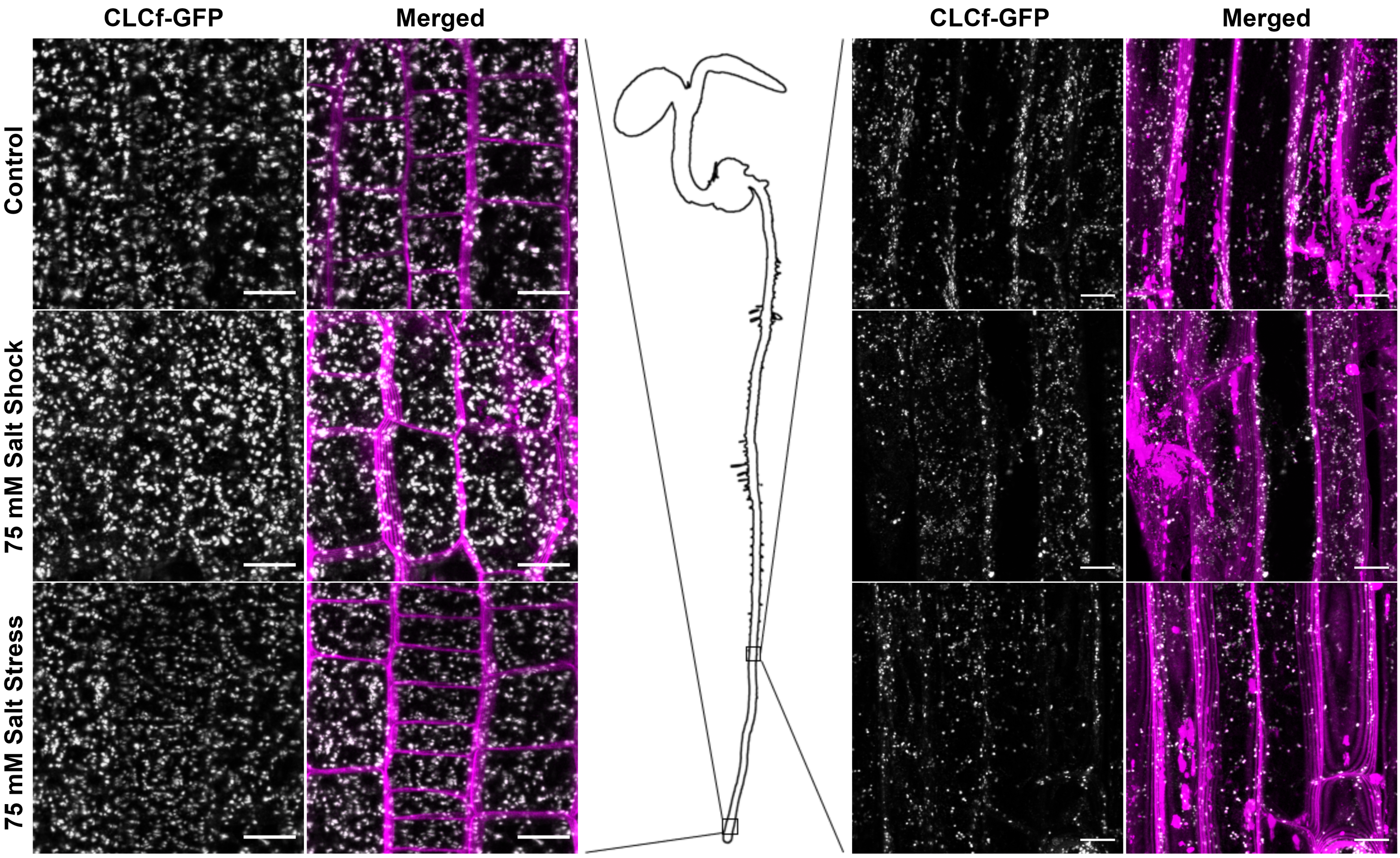

Supplementary Figure S1. CLCf-GFP localizes to puncta in root cells under control and salt stress conditions. Maximum intensity projections of CLSM image stacks of CLCf-GFP (white) and FM6-64 labelled PM (magenta) in root epidermal cells of both the elongation zone (left) and in mature root cells (right) after treatment with 75 mM NaCl for 6 hours (Salt Shock) or when constitutively grown on media containing 75 mM NaCl (Salt Stress). Scale bars = 10 µm.

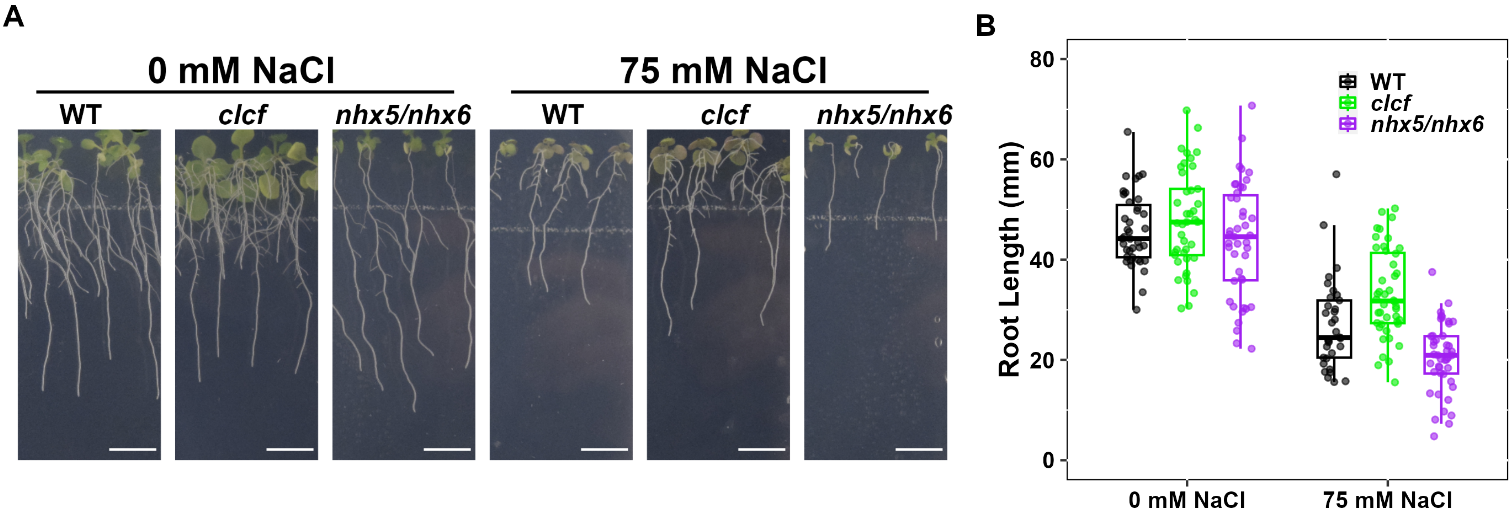

Supplementary Figure S2. CLCf is not required for maintaining root growth of seedlings during moderate salt stress. A-B) The primary root length of WT, clcf and nhx5/nhx6 mutant plants was measured after 2 weeks grown in media with or without 75 mM NaCl. The upper white line is part of the background equipment. Scale bars = 10 mm. For the boxplots, center line = median, box limits = upper and lower quartiles, whiskers = range to a maximum of 1.5x the interquartile range, points = individual data points.
